# Socio-emotional challenges and development of children left behind by migrant mothers

**DOI:** 10.7189/jogh.10.010806

**Published:** 2020-06

**Authors:** Xueqi Qu, Xi Wang, Xiaona Huang, KC Ashish, Yuning Yang, Yue Huang, Chunyi Chen, Yaqing Gao, Yinping Wang, Hong Zhou

**Affiliations:** 1 Department of Maternal and Child Health, School of Public Health, Peking University, Beijing, China; 2 Department of Epidemiology, Johns Hopkins Bloomberg School of Public Health, Baltimore, Maryland, USA; 3 Children’s Hospital of Philadelphia, Philadelphia, USA; 4 UNICEF China, Beijing, China; 5 Department of Women’s and Children’s Health, Uppsala University, Sweden

## Abstract

**Background:**

With great economic development and rapid urbanization in China, left-behind children whose parents migrate to big cities for job has become a large special population which requires more attention. The present study aims to explore the specific influence of migrant mothers on early child development, especially on social-emotional problems.

**Methods:**

The data of this study was obtained from a cross-sectional study in 8 counties of central and western rural China. Development status of 1880 children aged <60 months were assessed by Ages & Stages Questionnaire-Chinese Edition (ASQ) and the Ages and Stages Questionnaire: Social Emotional-Chinese Edition (ASQ: SE). Multivariate logistic regressions were used to analyze the association between being left behand by migrant mothers and developmental problems in various domains, while adjusting socio-demographic, socio-economic and perinatal co-variates, and effect modification analysis were conducted to explore the effect of age, gender and birth order.

**Results:**

Children left behind by migrant mothers were more likely to have overall suspected developmental delay (odds ratio (OR) = 1.24, 95% confidence interval (CI) = 1.13-1.35), developmental delay in personal social domain (OR = 1.55, 95% CI = 1.17-2.04) and socio-emotional delay compared with those living with their own mothers (OR = 1.49, 95% CI = 1.11-2.00) after adjusting for potential confounders. Additionally, girls increased the odds of social-emotional problems among children being left behind by migrating mother (*P* for interaction = 0.037).

**Conclusions:**

The study concluded that children left behind by migrant mothers were more likely to have suspected developmental delay compared with their peers living with mothers, especially on social emotional development. Future intervention is needed for this special population and should pay more attention to girls.

With reform and open-up during the past four decades, urban areas in China experience rapid social and economic development, and provide attractive job opportunities for labors in rural areas. According to data from National Bureau of Statistics of China, it is estimated that 180 million of rural population have migrated for seeking employment opportunities in the 3^rd^ quarter of 2017 [1]. The Chinese government administer residents, provide social services and allocate welfare according to “HUKOU”, which is similar with “an internal passport” and residents’ living address could be located by it [2]. Given that these migrant workers do not have “HUKOU” in the city where they work and live, their children are lack of access to public education in the cities, so many of them have to leave their children in the rural hometown with other family members, which are grouped as left-behind children [3]. Left-behind children refers to those below the age of 18 years who lived in their HUKOU location with one parents or other family members since one or both of their parents migrate to and live in other places for work [4]. All-China Women's Federation (ACWF) reported that there were about 23 million left-behind children below the age of 5 by 2010, increased rapidly by 7.47 million compared with 2005, and contributed 38.5% of 60 million left-behind children in rural China [5].

There is increasing body of literature concerning physical and mental health of this special population. Left-behind children face higher risk of slower physical growth compared with their peers not being left-behind [6]. It is well-documented that left-behind children have more mental and behavioral problems compared with general population of children: for example, depression, anxiety, severe psychological distress, alcohol consumption, and internet addiction [7-9]. However, while relationship between being left-behind and social-emotional problems is well documented during adolescence, much less is known regarding the association between socio-emotional development and left-behind children in early childhood. Socio-emotional development is a comprehensive concept widely used among preschool children, which refers to capability of communicating with social environment, dealing with social relationships and regulating emotion [10]. Delay in socio-emotional development in early childhood is related with emotional and behavioral problems in later childhood and even adolescence. Therefore, screening and early detection of socio-emotional problems is crucial for follow-up treatment and long-term mental health.

Accumulative evidence has shown that maternal characteristics, such as maternal depression, maternal cyclothymic temperament, and maternal education, play an important role on their children’s socio-emotional development [10,11]. But few studies have analyzed how absence of mother due to migration for work will affect child socio-emotional development. Therefore, the present study aimed to explore mother’s role on neurodevelopmental problems among <60 months left-behind children, especially socio-emotional development. Moreover, we compared two screening measurements: Ages and Stages Questionnaires and Ages and Stages Questionnaires: Social-Emotional on evaluating socio-emotional development.

## METHODS

### Participants

The data was collected from a cross-sectional survey in 8 rural counties of 4 provinces in central and western China in the year of 2016, Xinjiang, Qinghai, Jiangxi and Ningxia respectively. The annual income per capita in the project counties was lower than average income level in rural area of China in 2016: 8997 RMB (US$1297) vs 12 363 RMB (US$1782).

A multiple stage sampling method was applied to select the study population in 8 project counties, where the total population was 3 639 000 in 2016. First, 15 administrative villages per county and 2 nature villages for each administrative village were designated by random based on population proportional to size (PPS) method. Within each chosen nature village, the local doctor provided a list of households who have child under 5 years old. Finally, 10 households in each natural village were chosen by simple random sampling according to the list. The youngest child in each household and their caregivers were interviewed by face-to-face, and all the children received physical examination. This study was approved by the Peking University Health Science Center Institutional Review Board. Informed consent was obtained from each caregiver participating the survey.

### Measures

The caregivers were asked whether parents of the child live with the child or have lived and worked in other places for more than 6 months. Since the present study focus on important role of mother on child development, according to the answer on the question, children in the survey were dichotomized into two groups: living with mother now or mother being migrated for work.

The Chinese version of the “Ages and Stages Questionnaires (ASQ-C)” was used in the survey to evaluate child neurodevelopment [12,13]. ASQ-C has 21 versions according to various age interval from 1 to 66 months. The questionnaire is consisted of 30 items covering five developmental domains, that is, communication, fine motor, gross motor, problem solving and personal social. The answers for each question included “Yes”, “Sometimes” and “No”, corresponding to 10, 5, and 0 points. The sum of scores for six questions in each domain was calculated. Lower score represents worse neurodevelopment, and score of any domain less than two standard deviations below the mean area score for the Chinese reference group was considered to be positive screened. The validity of ASQ-C has already been explored. Compared to Bayley Scales of Infant Development (BSID-II), the sensitivity and specificity of ASQ-C were 85.00% and 84.26%, respectively [14].

The social-emotional development of the child was reported by the caregivers with the Chinese version of Ages and Stages Questionnaires: Social-Emotional (ASQ:SE-C) [15,16]. The ASQ:SE has 8 versions for different age groups, including 6, 12, 18, 24, 30, 36, 48, and 60 months, and those versions of the questionnaire consists of different number of items (from 19 to 33 items). ASQ:SE also divided as different domains, including self-regulation, compliance, communication, autonomy, affect, adaptive functioning and interaction with people. But the number of questions for the same domain is different in versions for different age groups, and it explains why different versions have unequal number of items. There are two kinds of answer to each specific question: the first kind of answer varied from ‘most of the time’, ‘sometimes’ or ‘rarely or never’, and 0, 5 and 10 points were given to each item accordingly; the second kind of answer is dichotomized as whether worrying about child’s appearance or not, and if parents choose “Yes”, and question will be graded 5 points additionally. Eventually total score was calculated, and higher score represents worse social-emotional development. Each version has its specific cut-off score. Those who have scores higher than the cut-off were considered as ‘at risk of socioemotional development delay’. The internal consistency of ASQ:SE-C was measured by Cronbach’s а, which ranged from 0.56-0.77 across various age interval, and the item reliability of ASQ:SE-C ranged from 94% to 96% [17]. The process of both questionnaires took about 15 minutes.

A self-designed structured questionnaire was applied in the survey, which referenced “Multiple indicator cluster survey (MICS) manual” published by the United Nations Children's Fund (UNICEF) [18]. Information including age, gender and birth order of child, gestational age, birthweight, delivery method, educational level of caregivers and household income was taken into consideration in the analysis. Except for gestational age and birthweight, other variables were categorical variables. The birth order was dichotomized as “1” or “2 and above”. The delivery method was coded as “cesarean section” “vaginal delivery” or “Missing”. In order to measuring household income level, we first calculated the annual net income of household by using total household income minus the expenses for the year. The type of household income included salary, income from part-time job, income from family agriculture production and government case subsidies. Household expenses included agricultural productive expenses (seeds, fertilizers, pesticides, etc.), living expenses (clothing, food, household appliance, etc.), health care expenses, and tax. Then we divided annual net income of household by the number of household members and obtained per capita net income of household. Eventually, the household income level was categorized into five levels based on the quintiles in the distribution of household per capita income in surveyed areas: poorest (lowest 20%), poor (21%-40%), middle (41%-60%), richer (61%-80%), richest (highest 20%). And the education level of caregivers was measured by five levels: illiterate, primary school, secondary school, college, and above.

### Data analysis

1880 children with completed information on ASQ and ASQ:SE measurement were included in final analysis. The distribution of left-behind children on socio-demographic characteristics were calculated, and tested by *t* test and χ^2^ test. Since we used PPS method in sampling, the cluster effect of the study design should be considered when estimating the prevalence of suspected developmental delay and the association between being left-behind by migrating mothers for work and suspected developmental delay. We used multi-level fixed logistics regression model in the analysis: the first stage is the administrative village and the second stage is the natural village. The predictor variable is child living with their own mother or not, and the dichotomized outcome variables include: total suspected developmental delay screened by ASQ, suspected developmental delay in each domain of ASQ, suspected socio-emotional developmental delay screened by ASQ:SE. Additionally, birth weight, gestational age, gender of child, age of child, birth order of child, education level of caregivers, delivery methods and household income were adjusting in the model. Children with missing values of above covariates were excluded in the adjusted model. Moreover, effect modification analysis was performed by adding interaction terms into logistics regression model, in order to explore modify effect of child’s age, gender and birth order. The strength of the association was estimated as odds ratios (ORs) and 95% confidence intervals (95% CI), and *P* for interaction was also reported. The control group in all models is child living with mothers. All statistical analyses were conducted using STATA 15 for Windows (Stata Corp, College Station, TX, USA), and statistical significance level was set at *P* < 0.05.

## RESULTS

In total, 1880 children were included in the present study, and 16.9% were left-behind with mother migrating for work. And of the 1880 children, 65.6% children whose mothers reported information in the interview, and 8.6% for fathers, 18.3% for grandmothers, 6.4% for grandfathers and 1.1% for other relatives. The basic characterisitics of participants were described in **Table 1**. There was a higher proportion of being the second and above child in the family among children with mother migrating for work, compared with children living with their mothers. 7.3% children with mother migrating for work were aged between 1-11 months, while the proportion of it among children living with mothers was 19.5%. In the group of children with mother migrating for work, more than half of caregivers were Illiteracy or graduated from primary school, and the proportion was relatively higher than that among children living with their mothers. As for income level, nearly 1 in 3 families with mother migrating for work ranked as middle level of household income, while 13.7% families with mother migrating for work could be considered as the richest.

**Table 1:** Basic characteristics of participants

	Total	Children living with mothers	Children left behind by migrant mothers	*P*-value
**Number (%)**	1880 (100)	1563 (83.1)	317 (16.9)	
**Gestational age weeks, mean (SD)**	39.2(1.6)	39.3(1.6)	39.1(1.5)	0.071
**Birthweight g, mean (range)**	3200(1000-5900)	3210 (1000-5900)	3147 (1650-4500)	0.042
**Age of child, months, n (%):**				<0.001
1-11	328 (17.5)	305 (19.5)	23 (7.3)	
12-23	504 (26.8)	414 (26.5)	90 (28.4)	
24-59	1048 (55.7)	844 (54.0)	204 (64.4)	
**Gender of child, n (%):**				0.053
Boys	1012 (53.8)	825 (52.8)	187 (59.0)	
Girls	868 (46.2)	738 (47.2)	130 (41.0)	
**Birth order of child, n (%):**				0.004
1	743 (39.5)	595 (38.1)	148 (46.7)	
≥2	1137 (60.5)	968 (61.9)	169 (53.3)	
**Caregiver’s education, n (%):**				<0.001
Illiteracy	247 (13.1)	139 (8.9)	108 (34.1)	
Primary	447 (23.8)	324 (20.7)	123 (38.8)	
Secondary	1044 (55.5)	963 (61.6)	81 (25.6)	
College and above	142 (7.6)	137 (8.8)	5 (1.6)	
**Method of delivery, n (%):**				0.305
Cesarean section	442 (23.7)	360 (23.3)	82(26.0)	
Vaginal delivery	1422 (76.3)	1188 (76.7)	234 (74.0)	
Missing	16	15	1	
**Income, n (%):**				<0.001
Poorest	411 (22.6)	347 (23.0)	64 (20.5)	
Poor	312 (17.1)	259 (17.2)	53 (16.9)	
Middle	406 (22.3)	302 (20.0)	104 (33.2)	
Richer	232 (12.7)	183 (12.1)	49 (15.7)	
Richest	460 (25.3)	417 (27.7)	43 (13.7)	
Missing	59	55	4	

SD – standard deviation

Being screened by ASQ, children with mother migrating for work have higher prevalence of total suspected developmental delay compared to their peers, but the difference is not statistically significant (26.8% vs 21.6%, *P* < 0.001) (**Table 2**). As for specific domains, children with mother migrating for work have significantly higher prevalence of communication delay (10.7% vs 7.5%, *P* < 0.05) and personal social problems (12.3% vs 7.4%, *P* < 0.05), and lower prevalence of problem solving problems (5.7% vs 7.4%, *P* < 0.05), while the prevalence of suspected developmental delay in other domains was similar between the two groups of children living with mothers or not. Being screened by ASQ:SE, children left behind by migrant mothers have higher prevalence of suspected social-emotional development delay compared to children living with mothers (33.1% vs 27.3%, *P* < 0.05). More importantly, the prevalence of social-emotional development delay screened by ASQ:SE was higher than prevalence of delay in personal social domains screened by ASQ in both two groups.

**Table 2:** Prevalence of suspected developmental delay in two groups (N = 1880)

	Children living with mothers	Children left behind by migrant mothers	*P*-value
Total suspected developmental delay	21.6	26.8	<0.001
Communication	7.5	10.7	0.004
Fine motor	6.5	6.6	0.893
Gross motor	7.2	6.9	0.612
Problem solving	7.4	5.7	0.011
Personal social	7.4	12.3	0.008
Social emotional	27.3	33.1	0.019

**Table 3** showed the association between being left behind by migrant mothers and suspected developmental delay. In the crude model, considering the ASQ screening, children left behind by migrant mothers were more likely to be screened positive for total suspected developmental delay, suspected developmental delay in domains of communication and personal social ability; were less likely to be screened positive for delay in problem solving domain. After adjusting various socio-demographic variables, children left behind by migrant mothers remained to be more likely to have total suspected developmental delay (OR = 1.24, 95% CI = 1.13-1.35), and suspected developmental delay in personal social ability (OR = 1.55, 95% CI = 1.17-2.04) remained, while the association between being left behind by migrant mothers and suspected developmental delay in domains of communication and problems solving ability disappeared. As for ASQ:SE, the significant association between being left behind by migrant mothers and social-emotional developmental delay existed in both crude and adjusted models (crude OR = 1.32, 95% CI = 1.07-1.62; adjusted OR = 1.49, 95% CI = 1.11-2.00).

**Table 3:** Association between developmental delay and being left behind by migrating mothers

	Percentage	Crude model (N = 1880)			Adjusted model* (N = 1618)		
	**OR**	**95% CI**	***P*-value**	**OR**	**95% CI**	***P*-value**
Total suspected developmental delay	22.6	1.33	1.27-1.39	<0.001	1.24	1.13-1.35	0.001
Communication	8.2	1.48	1.21-1.82	0.004	1.23	0.84-1.79	0.216
Fine motor	6.8	1.02	0.76-1.36	0.893	1.17	0.89-1.53	0.211
Gross motor	7.2	0.97	0.82-1.14	0.612	1.00	0.91-1.09	0.933
Problem solving	7.3	0.75	0.62-0.91	0.011	0.95	0.72-1.23	0.612
Personal social	8.1	1.77	1.25-2.49	0.008	1.55	1.17-2.04	0.010
Social emotional	28.3	1.32	1.07-1.62	0.019	1.49	1.11-2.00	0.018

CI – confidence interval, OR – odds ratio

*Adjusted for age, gender, birth order, gestational age, and birthweight of child, highest education level of caregivers, delivery method and household income.

The results of effect modification analysis were displayed in **Table 4**. As for age, the strong association between being left behind by migrant mothers and social-emotional developmental delay existed in the age group of 1-11 months (OR = 1. 77, 95% CI = 1.51-2.07) and 24-59 months (OR = 1.74, 95% CI = 1.56-1.95). The strength of the association also appeared strong in the group of being the second and above children in the family (OR = 1.89, 95% CI = 1.65-2.18). However, *P* for interaction shows that there is no statistical interaction of age and birth order on the association. Meanwhile, gender is shown to have strong influence on the association: girls who were left behind by migrant mothers are more likely to have suspected social-emotional developmental delay (OR = 2.67, 95% CI = 2.29-3.11), while the association is not significant among boys (*P* for interaction = 0.031)

**Table 4:** Effect modification of child’s age, gender and birth order on association between social emotional developmental delay and being left behind by migrating mothers.

	Children left behind by migrant mothers			
	**OR**	**95% CI**	***P*-value**	*P* for interaction*
**Age of child, months:†**				
1-11	1.77	1.51-2.07	<0.001	0.073
12-23	1.03	0.46-2.30	0.926	
24-59	1.74	1.56-1.95	<0.001	
**Gender of child:‡**				
Boys	1.00	0.50-2.00	0.988	0.031
Girls	2.67	2.29-3.11	<0.001	
**Birth order of child:§**				
1	1.08	0.57-2.03	0.777	0.077
≥2	1.89	1.65-2.18	<0.001	

CI – confidence interval, OR – odds ratio

*Assessed in logistic regression models with a product term between mother’s absence and stratifying variable (age/gender/birth order).

†Adjusted for gender, birth order, gestational age, and birthweight of child, highest education level of caregivers, delivery method and household income.

‡Adjusted for age, birth order, gestational age, and birthweight of child, highest education level of caregivers, delivery method and household income.

§Adjusted for age, gender, gestational age, and birthweight of child, highest education level of caregivers, delivery method and household income.

## DISCUSSION

Our results indicated that children with mother migrating for work were more likely to have suspected total developmental delay compared to their peers not being left behind, especially on socio-emotional developmental delay. The strength of this association is larger among left-behind girls compared with boys, which suggests that the left-behind girl is the population at risk and needs more attention. Moreover, we compared two developmental screening measurements: ASQ and ASQ:SE; and found that ASQ:SE has higher detectable rates on socio-emotional developmental delay compared to personal social domains of ASQ.

Our finding is similar with Jee et al study, which implies that combination of ASQ:SE and ASQ on screening socio-emotional development delay is more appreciated [19]. The differences of two questionnaires might explain the difference of detectable rate. ASQ is a comprehensive questionnaire to evaluate early child development, including domains of communication, fine motor, gross motor, problem solving and personal social. The personal social ability screened by ASQ actually partly reflects socio-emotional development, since it focuses on child’s ability to reflect on environmental stimulations. However, ASQ:SE is a specific questionnaire screening socio-emotional development by more questions on more aspects of socio-emotional development, including self-regulation, compliance, communication, autonomy, affect, adaptive functioning and interaction with people. The choice of two measurements should take feasibility into consideration. If the resource is limited, ASQ is more suitable to be primary screening measurement, since it reflects overall child neurodevelopment. If there is feasibility and the target population have high risk of socio-emotional development, we recommend apply ASQ and ASQ:SE at the same time to find out more cases.

The present study indicated that children with mother migrating for work were more likely to have overall suspected developmental problems. Until now, many studies have suggested effects of maternal parenting on child’s development, and many of them focused on prenatal maternal depression, which would influence parent-child interaction [20-22]. To our knowledge, our study first focused on early child development problems associating with mother migrating for work, a phenomenon which also induces lack of maternal parenting involvement. Moreover, our study suggested that comparing with other domains, these left behind children had higher risk of socio-emotional problems. It is consistent with several previous study which also reported higher risk of various mental health problems among left-behind children [23,24]. Zhao’s study emphasized that parent-child cohesion was associated with emotion adaptation of left-behind children [25]. There are several reasons why left-behind children have higher risk of socio-emotional problems. First, in Chinese family, the mother normally has the highest frequency of communicating with child among family members. Therefore, child without living with mother lose opportunity to learn and practice how to express their emotions. Second, animal studies also suggested that maternal separation can cause emotional alterations by inducing neuroinflammation [26].

The previous studies mainly focused on school-age children and adolescents and most of them reported higher prevalence or risk of depression, anxiety or behavioral problems [27,28]. Our study provided evidence for early child development among left-behind children, which might suggest the effect of being left-behind begins at early life of children and might be stable or worse over years. Therefore, in order to reduce serious mental and behavioral problems among left-behind children, we should take action in early childhood.

Our study shows that girls were more sensitive to the association in comparison to their boy counterparts. But previous studies among left-behind children mentioned that being left behind is more likely to increase the risk of alcohol and internet addiction among boys rather than girls [7,9]. This inconsistence might be due to different outcomes of interest in different studies: boys are more likely to cope their socio-emotional problems by risky behaviors during adolescence, which might not equal to high prevalence of socio-emotional problems. Additionally, considering traditional attitude of valuing males and boys much more than females and girls in many rural areas of China, the emotional support from family for girls might be less than that for boys. Therefore, maternal support is much important for girls compared to boys.

Admittedly, our study has several limitations. First, although we interviewed primary caregivers, but the questionnaire lacks detailed information on parenting and communication with child, for example how many hours the primary caregiver spends companying with the child every day and how many hours of effective communication. However, the types of primary caregivers and the difference in quality of parenting might also influence early child development. For 317 children with migrating mothers, more than 60% of them were in custody of their grandmothers, others were taken care of by fathers, grandfathers and other relatives. We further compared the prevalence of different types of developmental delay across groups of different types of caregivers in Table S1 in the **Online Supplementary Document**. The prevalence of total suspected developmental delay, delay in communication domain, delay in personal social domain and delay in social emotional domain among children living with their mothers were lower than that among children cared by their fathers, grandparents and other relatives, respectively, and the results were consistent with that of the comparisons between children with migrating mothers and without migrating mothers. The findings support our conclusion on mothers positive and significant role in improving child neurodevelopment, and also support our decision to treat the three primary caregiver groups (ie, fathers, grandmothers, and grandfathers) as a homogeneous population in our comparison group. Moreover, as shown in supplementary table, generally children cared by grandmothers had better performance in neurodevelopment than those who cared by fathers and grandfathers. However, given the limited sample size, we were unable to explore the influence on types of primary caregivers on the association between mothers’ migration and child neurodevelopment. Future study is supposed to explore further on influence of different primary caregivers on child neurodevelopment.

Second, there is lack of the detailed description on duration, reason or history of migration so that the current study is unable to explore the influence of extent of migration on above associations. But the question when the mother migrates for work (how old the child is at that time) was asked in the interview, and the duration of migration was calculated based on this question briefly. The median duration was 17.2 months and the range were from 0.17 months to 56.3 months. Our interviewers emphasized in the interview that the meaning of migrating for work in our study is the situation where the mothers will not eat and live at home every day. Therefore, our study distinguished the daily migrating mothers (commuters) and those who migrate for longer periods. But we were still unable to measure the “dose” of mother’s migration and explore dose relationship between being left behind and social emotional competence. Moreover, some mothers might migrate purposely trying to defer them from the child, which can overestimate the negative influence of mother migration on child neurodevelopment. Given that the interview asked if the mother migrate to other places for work and emphasized the purpose as working with participants, majority of mothers with the response of yes should be considered as migrating for more job opportunities and making more money. But it is true that we did not have more information on reason of migration.

Third, the quality of ASQ-C and ASQ: SE-C should be taken into consideration when interpreting the result. ASQ:SE-C has relatively low internal consistency. Generally, Cronbach’s α above 0.7 can be interpreted as good internal consistency. But the alphas of ASQ:SE-c ranged from 0.56 to 0.77 in different age intervals. According to the study which analyzed to evaluate the psychometric properties of ASQ:SE-C, the alphas of questionnaires for different age groups are different [17]. Older age intervals had better internal consistency compared with younger age intervals, and might be explained by more items in questionnaires for older age intervals. Additionally, compared with Bayley [17], validity of ASQ-C is not very good, and both the sensitivity and specificity of ASQ-C did not achieve 90% and above. It is admitted that given the fair reliability and validity, ASQ and ASQ:SE should be applied as screening tool instead of diagnosis tool and should be interpreted carefully [14]. However, the major advantage of ASQ and ASQ:SE is cost-effective, which means that it is suitable for wide-scale screening in resource-limited areas, like the western rural areas of China in our study. Fourth, since we did not mask staffs who measured child neurodevelopment to the maternal status strictly, the staffs might be influenced by preconception on children not living with mothers. But actually, the interview on general information and measurement of ASD and ASD-SE were conducted separately by two groups of staffs. Thus, the effect of not blinding might have influence on the association but the influence is relatively limited.

## CONCLUSION

To conclude, children left behind by migrating mothers were more likely to have overall suspected developmental delay and delay in social emotional ability. Additionally, girls were more likely to be influenced by above associations compared to boys. Thus, in order to improve growth and development of left-behind children, the interventions focusing on preschool stage and socio-emotional development could be effective. Moreover applying ASQ and ASQ:SE combinedly is essential for screening overall developmental delay and socio-emotional developmental delay in population with high risk.

## Additional material

Online Supplementary Document
