# Supplementary material for: Socio-emotional challenges and development of children left behind by migrant mothers
Source: J Glob Health. 2020 Apr 15;10(1):010806. doi: 10.7189/jogh.10.010806 (PMC7182358; doi:10.7189/jogh.10.010806)
Supplement: Online Supplementary Document [file jogh-10-010806-s001.pdf]

Supplementary Table 1 Prevalence of suspected developmental delay across different primary caregivers groups (N=1880)

	Children living with mothers	Children with migrating mothers and living with other primary caregivers				
		Fathers	Grandmothers	Grandfathers	Others[1]	*P* value
N(%)	1563(83.1)	25(1.3)	205(10.9)	81(4.3)	6(0.3)	
Total Suspected Developmental Delay	21.6%	28.0%	22.9%	35.8%	33.3%	0.005
Communication	7.5%	12.0%	10.7%	9.9%	16.7%	0.043
Fine motor	6.5%	4.0%	5.4%	9.9%	16.7%	0.018
Gross motor	7.2%	8.0%	6.3%	6.2%	33.3%	0.047
Problem solving	7.4%	12.0%	4.9%	4.9%	16.7%	0.018
Personal social	7.4%	16.0%	10.2%	17.3%	0	0.002
Social emotional	27.3%	44.0%	30.2%	37.0%	33.3%	0.095

1
